# Mechanism-Dependent Effects of the α5 GABA_A_ Receptor Negative Allosteric Modulator Basmisanil on Spatial Memory

**DOI:** 10.3390/biom16101429

**Published:** 2026-10-02

**Authors:** Miruna Valeria Moraru, Aurelian Zugravu, Aurelia Cristiana Barbu, Smaranda Stoleru, Cristina Isabel Viorica Ghiță, Adrian-Florentin Dragomir, Andrei Stoian, Laurentiu-Nicolae Ciuca, Dragos Constantin Lunca, Clara Maria Stoleru, Oana Andreia Coman, Ion Fulga

**Affiliations:** 1 Faculty of Medicine, Pharmacology, Clinical Pharmacology and Pharmacotherapy Discipline, “Carol Davila” University of Medicine and Pharmacy, 050474 Bucharest, Romania; miruna-valeria.moraru@drd.umfcd.ro (M.V.M.); aurelian.zugravu@umfcd.ro (A.Z.); isabel.ghita@umfcd.ro (C.I.V.G.); adrian-florentin.dragomir@drd.umfcd.ro (A.-F.D.); andrei.stoian@umfcd.ro (A.S.); nicolae.ciuca@umfcd.ro (L.-N.C.); dragos.lunca@umfcd.ro (D.C.L.); oana.coman@umfcd.ro (O.A.C.); ion.fulga@umfcd.ro (I.F.); 2 Faculty of Medical Engineering, National University of Science and Technology POLITEHNICA Bucharest, 060042 Bucharest, Romania; clara_maria.stoleru@stud.fim.upb.ro

**Keywords:** basmisanil, α5 GABA_A_ receptor, negative allosteric modulator, spatial memory, Y-maze, diazepam, trihexyphenidyl, lipopolysaccharide, neuroinflammation, cognitive impairment

## Abstract

Cognitive impairment may arise through multiple pathophysiological mechanisms, including altered GABAergic neurotransmission, cholinergic dysfunction, and neuroinflammation. This study investigated whether basmisanil, a selective negative allosteric modulator of α5-containing GABA_A_ receptors, attenuates spatial memory impairment induced by diazepam, trihexyphenidyl (THP), or lipopolysaccharide (LPS). Adult male Wistar rats were assigned to three experimental models of memory impairment, each comprising four treatment groups (control, inducing agent, basmisanil, and inducing agent plus basmisanil; *n* = 7/group). Spatial memory was assessed using the two-trial Y-maze paradigm. The primary outcome (Time Index U) was analyzed using two-way factorial general linear models with sensitivity analyses. Significant interactions between the inducing agent and basmisanil were observed in the diazepam and LPS models, indicating attenuation of memory impairment following basmisanil coadministration, whereas no significant interaction was detected in the THP model. The largest estimated recovery effect was observed in the diazepam model, followed by the LPS model and the THP model, although cross-model comparisons were not statistically significant. Basmisanil alone did not enhance baseline memory performance. These findings indicate that the behavioral effects of α5-containing GABA_A_ receptor modulation are mechanism-dependent and support further investigation of selective α5 negative allosteric modulators for benzodiazepine-associated memory impairment.

## 1. Introduction

Memory impairment is a hallmark of numerous neurological and neuropsychiatric disorders and represents one of the major determinants of functional disability and reduced quality of life. It can arise through multiple pathophysiological mechanisms, including alterations in GABAergic neurotransmission, cholinergic dysfunction, and neuroinflammation [1]. Although these mechanisms differ considerably, they ultimately disrupt hippocampal-dependent memory processing and contribute to impaired memory performance [2]. Understanding the relative contribution of these pathways is important for developing targeted therapeutic strategies capable of improving memory while minimizing adverse effects [3]. Among these mechanisms, GABAergic neurotransmission has attracted particular attention because of its critical role in regulating hippocampal information processing and memory formation.

Among the GABA_A_ receptor subtypes, receptors containing the α5 subunit have received considerable attention because of their predominant expression within the hippocampus and their role in learning and memory [4]. Unlike α1-containing GABA_A_ receptors, which primarily mediate sedative and amnestic effects, α5-containing GABA_A_ receptors contribute to tonic inhibitory neurotransmission within hippocampal circuits involved in learning and retrieval [5]. Experimental studies have consistently demonstrated that pharmacological modulation of α5-containing GABA_A_ receptors influences memory performance, making this receptor subtype a target for cognitive enhancement [6,7,8,9]. In particular, selective negative allosteric modulation (NAM) of the α5 GABA_A_ receptor has been proposed as a potential therapeutic strategy for improving memory without interfering with the anxiolytic or anticonvulsant effects mediated by other GABA_A_ receptor subtypes [10,11,12].

Basmisanil (RG1662) is a highly selective negative allosteric modulator of α5-containing GABA_A_ receptors that has shown procognitive effects in several preclinical models and subsequently progressed to clinical evaluation. It has been investigated primarily in conditions associated with cognitive dysfunction, including Down syndrome and other neurodevelopmental disorders [11,12,13]. Although clinical efficacy has been more limited than initially anticipated, its high receptor selectivity and favorable pharmacological profile have established basmisanil as an important experimental tool for investigating the role of α5-containing GABA_A_ receptors in cognition [13]. Although α5 receptor modulation has been extensively investigated in GABAergic models of cognitive dysfunction, considerably less is known about its ability to improve memory deficits arising from distinct neurobiological mechanisms, such as cholinergic dysfunction or neuroinflammation [13].

To investigate this knowledge gap, the present study employed three complementary experimental models of memory impairment in the Y-maze. Benzodiazepines are widely used for their anxiolytic, sedative, anticonvulsant, and myorelaxant effects; however, impairment of learning and memory remains one of their best-recognized cognitive adverse effects [14,15,16]. Diazepam was selected as a pharmacological model of benzodiazepine-induced amnesia because its non-selective modulation of GABA_A_ receptors includes activation of α5-containing receptors, which are strongly implicated in memory impairment [17]. Trihexyphenidyl (THP), a muscarinic receptor antagonist, produces reversible cognitive impairment through antagonism of central muscarinic acetylcholine receptors and is widely used as an experimental model of cholinergic dysfunction; therefore, it was selected to induce anticholinergic memory impairments [18]. Lipopolysaccharide (LPS), a bacterial endotoxin, activates the innate immune response through Toll-like receptor 4 (TLR4), promoting the release of pro-inflammatory cytokines and inducing neuroinflammation [19]. Accordingly, LPS was used to induce neuroinflammation-associated memory impairment [20,21,22].

By comparing these three mechanistically distinct models within an identical behavioral and statistical framework, the present study provides a systematic evaluation of whether the cognitive effects of α5 GABA_A_ receptor modulation are mechanism-dependent or represent a more general strategy for improving memory impairment [23,24].

The aim of the present study was to determine whether basmisanil attenuates spatial memory impairment induced by diazepam, trihexyphenidyl, or LPS in the Y-maze and to compare the magnitude of its effects across three experimental models of memory impairment.

## 2. Materials and Methods

A total of 84 adult male Wistar rats supplied by the animal breeding facility of the “Carol Davila” University of Medicine and Pharmacy, Bucharest, Romania, were used in this study. Animals were randomly assigned to one of three experimental models, each comprising four experimental groups (*n* = 7 per group). Model 1 included a control group, a diazepam group (4 mg/kg), a basmisanil group (10 mg/kg), and a diazepam + basmisanil group (4 + 10 mg/kg). Model 2 included a control group, a trihexyphenidyl group (10 mg/kg), a basmisanil group (10 mg/kg), and a trihexyphenidyl + basmisanil group (10 + 10 mg/kg). Model 3 included a control group, an LPS group (0.5 mg/kg), a basmisanil group (10 mg/kg), and an LPS + basmisanil group (0.5 + 10 mg/kg). Each animal participated in only one experimental protocol.

Rats were housed individually under controlled temperature and humidity, with a 12 h light/dark cycle, and provided with standard laboratory chow and water ad libitum. Dose selection was based on previously published pharmacological studies. Testing conditions were applied consistently across all groups, and behavioral testing was performed by the same investigator throughout the study. Animals were assessed in an alternating sequence to minimize potential bias related to spatial preference and odor cues.

All drugs were administered intraperitoneally (i.p.), and injection volumes were adjusted according to body weight. Diazepam (commercially available injectable solution, 5 mg/mL) was administered at 4 mg/kg [25,26]. Trihexyphenidyl (Romparkin^®^, 2 mg tablets, Terapia, Cluj-Napoca, Romania) was administered at 10 mg/kg. Lipopolysaccharide (LPS from *Escherichia coli* O55:B5; Sigma-Aldrich/Merck, St. Louis, MO, USA; catalog no. L2880) was administered at 0.5 mg/kg [21,27,28]. Basmisanil (RG1662, MedChemExpress, Monmouth Junction, NJ, USA) was administered at 10 mg/kg [13]. The control groups received the corresponding vehicle (DMSO). For the diazepam and THP co-administration groups, basmisanil and the inducing agent were mixed immediately before administration and delivered as a single i.p. injection. In the LPS model, LPS was administered 24 h before behavioral testing, followed by basmisanil administered 2 h before the acquisition phase (T1). This design ensured sufficient time for the development of LPS-induced neuroinflammation before administration of basmisanil and subsequent behavioral testing [29,30].

Spatial memory performance was evaluated using the Y-maze model [31]. In the diazepam and THP experiments, treatments were administered 2 h before the acquisition phase (T1). In the LPS experiment, LPS was administered 24 h before T1, whereas basmisanil was administered 2 h before T1. During T1, one arm of the maze was blocked, allowing exploration of the start (S) arm and one open (O) arm. Time spent in the open arm during T1 was recorded specifically to verify that exploratory activity during the acquisition phase did not differ between groups before the recall test. These measurements were not considered memory outcomes but served as baseline exploratory controls. Twenty-four hours later (T2), the previously blocked arm was opened, allowing exploration of both the previously known (K) arm and the previously unknown (U) arm. Animals were allowed to explore the maze in both phases for 5 min. Behavioral scoring was performed manually by direct observation by the same investigator throughout the study. No automated video-tracking software was used. Entry-based measures were analyzed as secondary behavioral outcomes to complement interpretation of the primary memory endpoint. The maze was cleaned between animals to minimize the influence of olfactory cues.

Statistical analyses were performed using IBM SPSS Statistics for Windows, version 29.0 (IBM Corp., Armonk, NY, USA), and statistical significance was defined as *p* < 0.05. The results were interpreted primarily based on effect estimates, 95% confidence intervals (CIs), and *p* values. The primary outcome was the Time Index for the U arm (Time Index U). Secondary outcomes included the time spent in the U and K arms, the proportion of entries into the U arm, and the total number of arm entries during T2. A value of 50% indicated equal exploration of the U and K arms, whereas values greater than 50% indicated a temporal preference for the U arm. Within each experimental model, Time Index U was analyzed using a 2 × 2 factorial general linear model including the inducing agent, basmisanil treatment, and their interaction. Type III sums of squares were used. Estimated marginal means (EMMs) and their 95% confidence intervals were calculated for each treatment combination. For each effect, the F statistic, degrees of freedom, *p* value, and partial eta squared (ηp^2^) were reported. For relevant treatment contrasts, mean differences, 95% confidence intervals, Holm-adjusted *p* values, and Hedges’ g effect sizes were reported. To evaluate whether the effect of basmisanil differed among the diazepam, trihexyphenidyl, and LPS models, a pooled 3 × 2 × 2 factorial general linear model (GLM) was performed. Sensitivity analyses using complementary parametric and non-parametric methods, bootstrap confidence intervals, and influence diagnostics were conducted to assess the robustness of the primary findings.

## 3. Results

### 3.1. Effects of Diazepam and Basmisanil on Spatial Memory Performance in the Y-Maze

Exploratory behavior during the acquisition phase (T1) was evaluated to exclude baseline differences that could influence subsequent recall performance. No significant differences were observed in the time spent exploring the open (O) arm among the experimental groups (Table 1).

Two-way factorial analysis revealed a significant main effect of basmisanil (F(1,24) = 13.49, *p* = 0.001, ηp^2^ = 0.360) and a significant diazepam × basmisanil interaction (F(1,24) = 19.79, *p* < 0.001, ηp^2^ = 0.452), whereas no significant main effect of diazepam was observed (F(1,24) = 1.71, *p* = 0.203) (Table 2).

For the primary outcome (Time Index U), estimated marginal means were 50.87% in the control group, 39.51% in the diazepam group, 49.34% in the basmisanil group, and 55.54% in the diazepam + basmisanil group (Table 3). Diazepam reduced preference for the previously unknown arm, whereas coadministration of basmisanil restored Time Index values to levels comparable with those of the control group.

**Figure 1:**
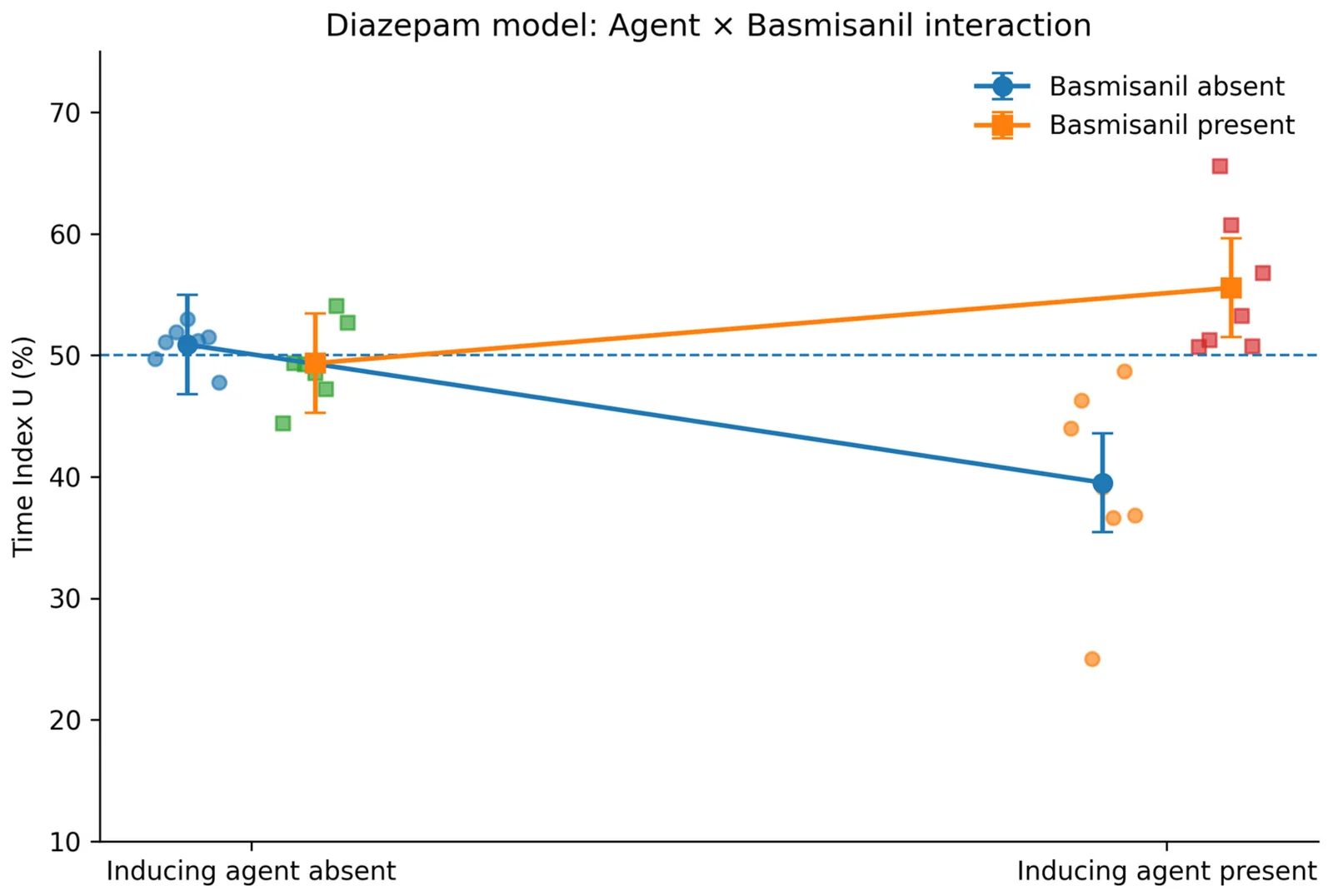
Interaction between diazepam and basmisanil on Time Index (%) in the Y-maze. Note. Points represent individual animals. Symbols and connecting lines represent estimated marginal means, with 95% confidence intervals. The dashed horizontal line indicates the 50% chance level.

A significant diazepam × basmisanil interaction was observed (Figure 1). Time Index values increased in animals receiving diazepam plus basmisanil compared with diazepam alone, whereas basmisanil alone produced values comparable to those of the control group.

Secondary outcome measures showed a similar descriptive pattern. Animals receiving diazepam spent less time in the U arm than controls, whereas the diazepam + basmisanil group exhibited the highest exploration time of the U arm. Measures based on the number of entries showed smaller differences and were therefore considered supportive rather than primary evidence for the observed behavioral effects.

Sensitivity analyses were consistent with the primary analysis and supported the robustness of the observed findings in the diazepam model across complementary statistical approaches.

### 3.2. Effects of Trihexyphenidyl and Basmisanil on Spatial Memory Performance in the Y-Maze

Exploratory behavior during the acquisition phase (T1) was evaluated to exclude baseline differences that could influence subsequent recall performance. No significant differences were observed in the time spent exploring the O arm among the experimental groups (Table 4).

Two-way factorial analysis revealed a significant main effect of THP (F(1,24) = 8.02, *p* = 0.009, ηp^2^ = 0.251), whereas neither the main effect of basmisanil (F(1,24) = 0.09, *p* = 0.772, ηp^2^ = 0.004) nor the trihexyphenidyl × basmisanil interaction (F(1,24) = 0.68, *p* = 0.419, ηp^2^ = 0.027) was statistically significant (Table 5).

For the primary outcome (Time Index U), estimated marginal means were 53.29% in the control group, 36.35% in the THP group, 50.84% in the basmisanil group, and 41.52% in the THP + basmisanil group (Table 6). THP reduced preference for the previously unknown arm compared with controls, whereas coadministration of basmisanil produced only a modest increase in Time Index values.

**Figure 2:**
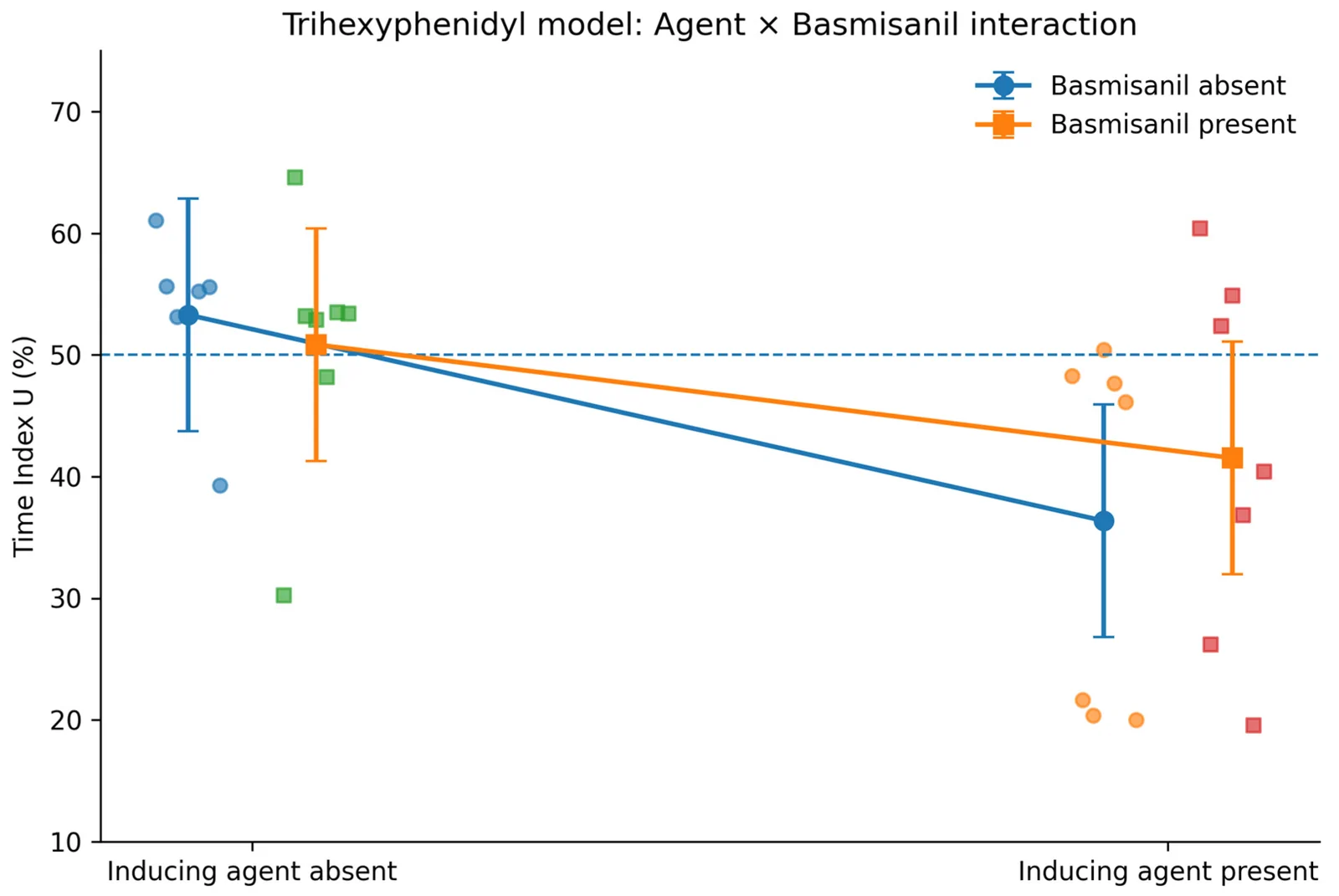
Interaction between THP and basmisanil on Time Index (%) in the Y-maze. Note. Points represent individual animals. Symbols and connecting lines represent estimated marginal means, with 95% confidence intervals. The dashed horizontal line indicates the 50% chance level.

No significant THP × basmisanil interaction was observed (Figure 2). Time Index values remained lower in the THP + basmisanil group than in controls and were similar to those observed in the THP group.

Secondary outcome measures showed a similar descriptive pattern. Animals receiving THP spent less time in the U arm than controls, whereas coadministration of basmisanil resulted in only a limited improvement. Measures based on the number of entries showed smaller differences and were therefore considered supportive rather than primary evidence for the observed behavioral effects.

Sensitivity analyses were consistent with the primary analysis and supported the robustness of the observed findings across complementary statistical approaches.

### 3.3. Effects of Lipopolysaccharide and Basmisanil on Spatial Memory Performance in the Y-Maze

Exploratory behavior during the acquisition phase (T1) was evaluated to exclude baseline differences that could influence subsequent recall performance. No significant differences were observed in the time spent exploring the O arm among the experimental groups (Table 7).

Two-way factorial analysis revealed a significant LPS × basmisanil interaction (F(1,24) = 6.91, *p* = 0.015, ηp^2^ = 0.224), whereas neither the main effect of LPS (F(1,24) = 2.30, *p* = 0.142, ηp^2^ = 0.088) nor the main effect of basmisanil (F(1,24) = 0.59, *p* = 0.448, ηp^2^ = 0.024) reached statistical significance (Table 8).

For the primary outcome (Time Index U), estimated marginal means were 53.58% in the control group, 36.24% in the LPS group, 45.81% in the basmisanil group, and 50.46% in the LPS + basmisanil group (Table 9). LPS reduced preference for the previously unknown arm compared with controls, whereas coadministration of basmisanil increased Time Index values toward those observed in the control group.

**Figure 3:**
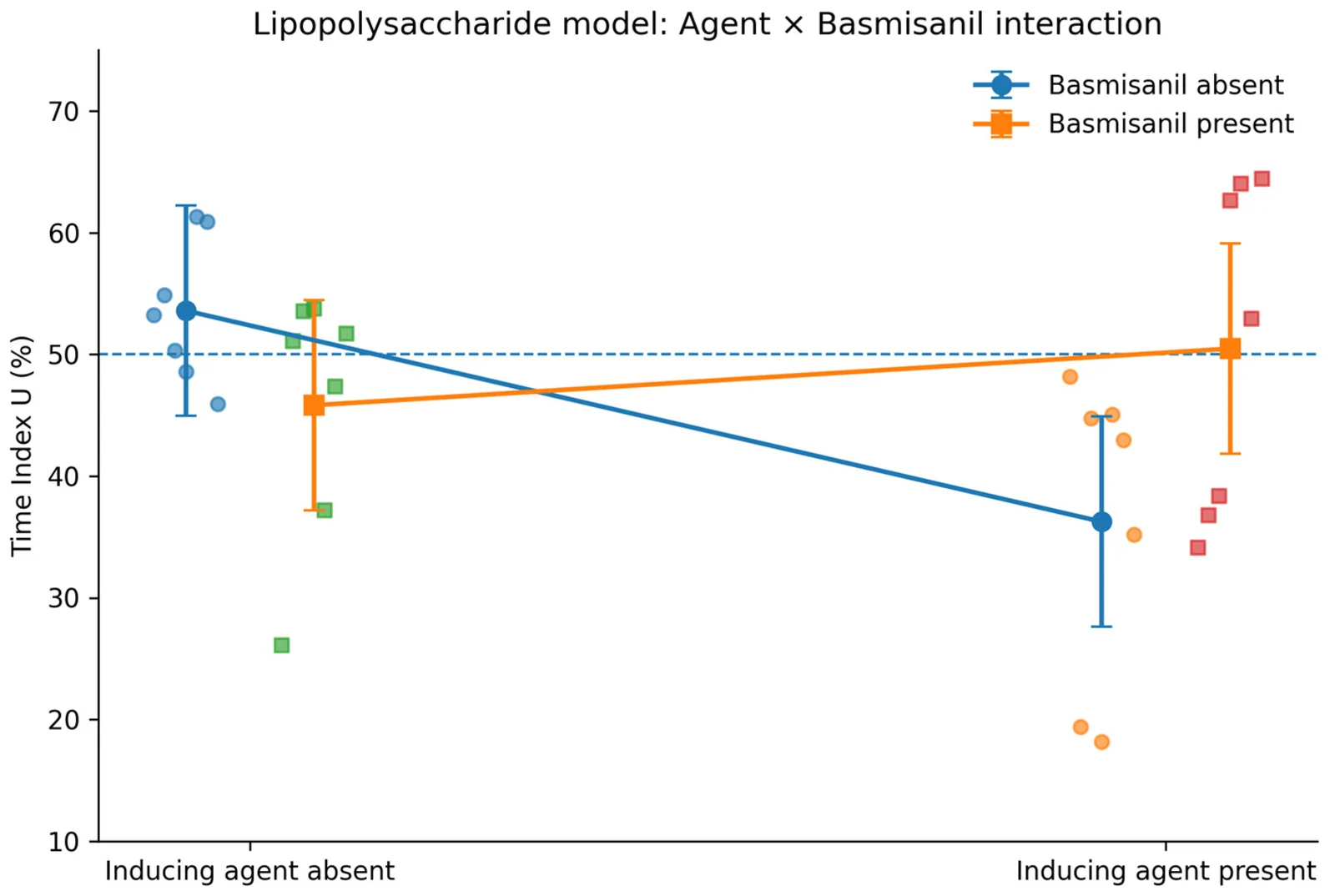
Interaction between LPS and basmisanil on Time Index (%) in the Y-maze. Note. Points represent individual animals. Symbols and connecting lines represent estimated marginal means, with 95% confidence intervals. The dashed horizontal line indicates the 50% chance level.

A significant LPS × basmisanil interaction was observed (Figure 3). Time Index values increased in the LPS + basmisanil group compared with the LPS group and approached those observed in control animals.

Secondary outcome measures showed a similar descriptive pattern. Animals receiving LPS spent less time in the U arm than controls, whereas coadministration of basmisanil increased exploration of the U arm. Measures based on the number of entries showed smaller differences and were therefore considered supportive rather than primary evidence for the observed behavioral effects.

Sensitivity analyses were consistent with the primary analysis and supported the robustness of the observed findings across complementary statistical approaches.

### 3.4. Comparison of Basmisanil Recovery Effects Across Models

**Figure 4:**
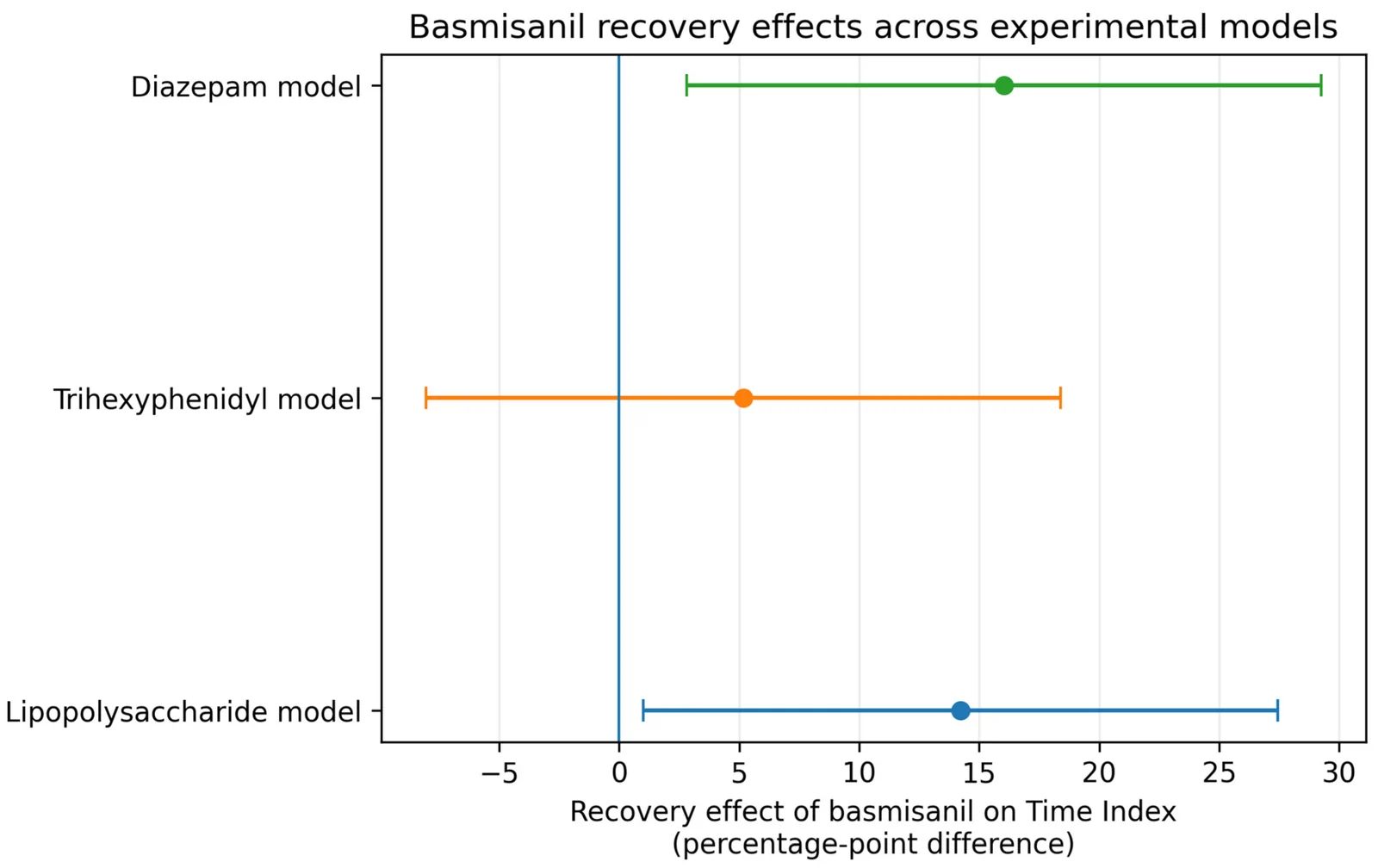
Basmisanil recovery effects across experimental models. Note. Positive values indicate a higher Time Index in the agent + basmisanil group than in the corresponding agent group. Error bars represent 95% confidence intervals.

To compare the magnitude of basmisanil-induced recovery across the three experimental models, recovery effects (Δ) were estimated as the difference between the agent + basmisanil group and the corresponding agent alone group (Table 10; Figure 4).

The largest estimated recovery effect was observed in the diazepam model (Δ = 16.04 percentage points; Hedges’ *g* = 2.17), followed by the LPS model (Δ = 14.22 percentage points; Hedges’ *g* = 1.01), whereas the smallest effect was observed in the THP model (Δ = 5.17 percentage points; Hedges’ *g* = 0.32). Recovery effects were greater in the diazepam and LPS models than in the THP model, although confidence intervals showed substantial overlap (Figure 4).

Planned contrasts confirmed that each inducing agent significantly reduced Time Index U compared with its corresponding control group (Table 11).

**Figure 5:**
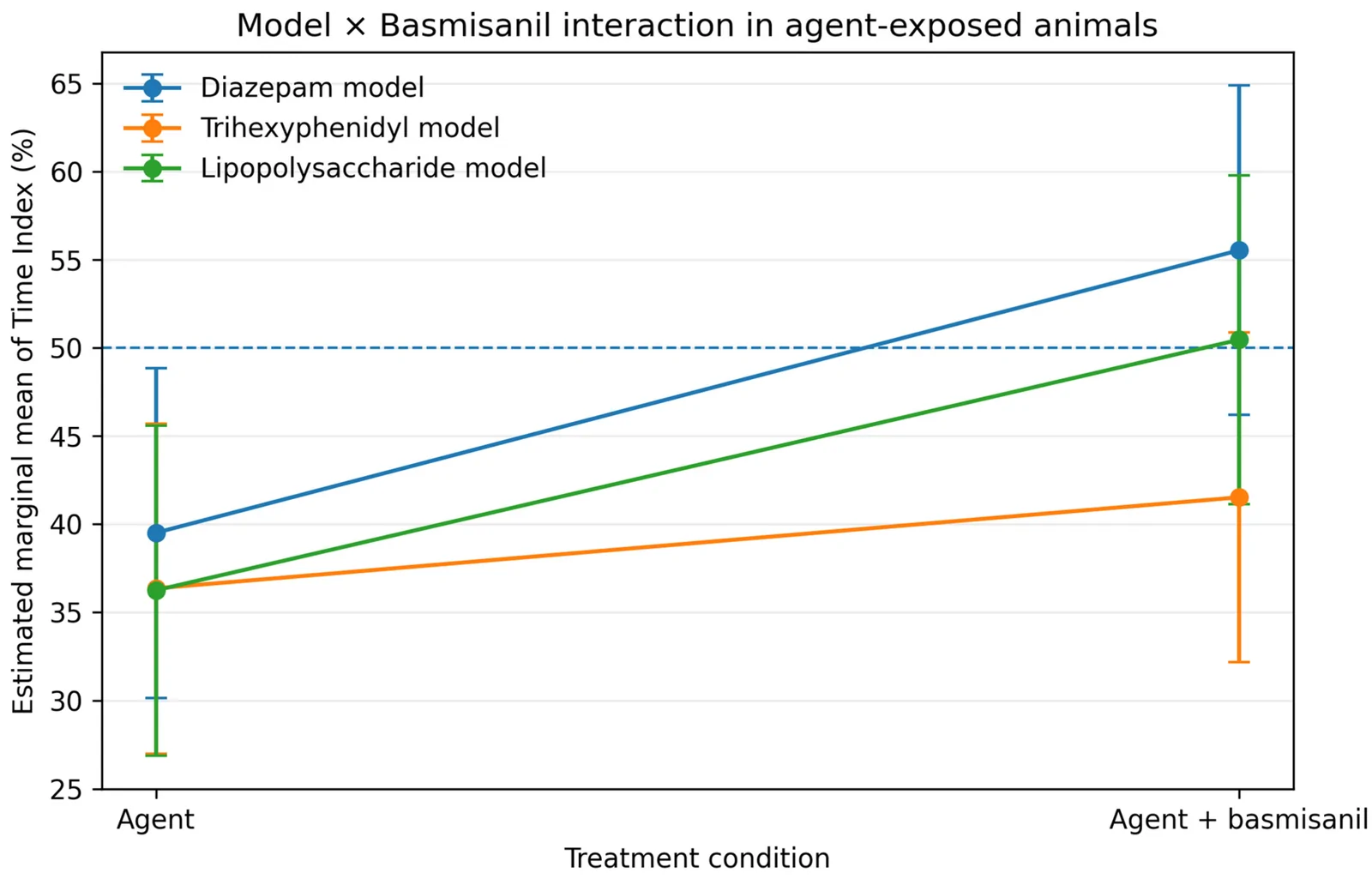
Model × basmisanil interaction in agent-exposed animals. Note. Points represent estimated marginal means, and error bars indicate 95% confidence intervals. The dashed horizontal line represents the 50% chance level of the Time Index.

Estimated marginal means further illustrated distinct response patterns across the experimental models (Figure 5). Coadministration of basmisanil markedly increased the Time Index in the diazepam model, produced only a modest change in the THP model, and showed partially restored Time Index values in the LPS model. Despite these descriptive differences, the pooled 3 × 2 × 2 GLM did not demonstrate a significant Model × Agent × Basmisanil interaction (Figure 5).

## 4. Discussion

Rather than exhibiting a uniform procognitive effect, basmisanil produced distinct behavioral responses depending on the experimental mechanism responsible for memory impairment.

The present study extends our group’s ongoing systematic research program investigating pharmacological modulation of cognition across complementary experimental models of memory impairment using a standardized Y-maze protocol. Previous studies employed the same behavioral and analytical framework to investigate cholinergic modulation in healthy animals and in models of cholinergic dysfunction and neuroinflammation, thereby establishing a consistent experimental platform for direct comparison of pharmacological interventions [20,21,22].

Building on this experimental platform, the present study investigated whether basmisanil, a selective negative allosteric modulator of the α5 subunit of the GABA_A_ receptor, could attenuate spatial memory impairment induced by three distinct experimental models: diazepam-induced amnesia, THP-induced cholinergic dysfunction, and LPS-induced neuroinflammation. By applying an identical behavioral protocol, experimental design, and statistical framework across all three models, the present study enabled a direct comparison of the recovery effect of basmisanil under different mechanisms of cognitive impairment. This comparative approach extends previous studies that have primarily evaluated α5 receptor modulation within a single experimental model and provides broader insight into the mechanism-dependent contribution of α5-containing GABA_A_ receptors to hippocampal-dependent memory [32,33,34,35].

**Figure 6:**
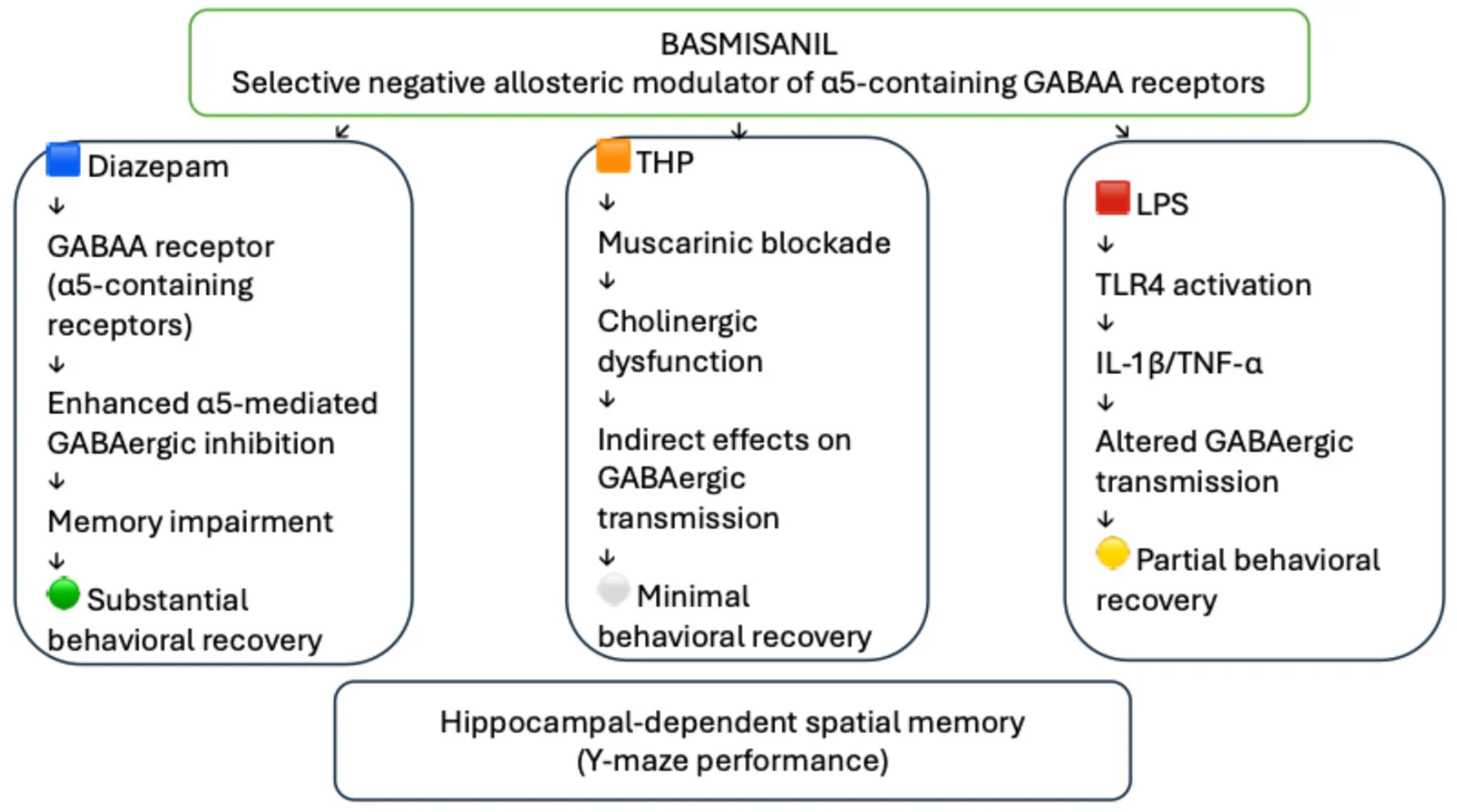
Proposed mechanisms underlying the model-dependent behavioral effects of basmisanil. The proposed mechanisms integrate the behavioral findings of the present study with previously published evidence and should be interpreted as a conceptual framework rather than direct mechanistic proof.

Planned contrasts confirmed that each inducing agent significantly reduced Time Index U relative to its corresponding control group, validating the three experimental models before evaluation of the basmisanil treatment effect. Although all three experimental models produced spatial memory impairment, the behavioral response to basmisanil differed according to the mechanism by which memory impairment was induced. Therefore, the individual models should be interpreted primarily based on their respective factorial analyses, while the cross-model comparison should be regarded as descriptive. In the diazepam model, a significant diazepam × basmisanil interaction and the highest estimated recovery effect supported selective attenuation of benzodiazepine-induced memory impairment. In contrast, no significant interaction was observed in the THP model, suggesting that cholinergic dysfunction may rely less on α5-mediated inhibitory mechanisms. In the LPS model, a significant interaction indicated partial improvement of spatial memory performance, although this effect appeared less robust than that observed in the diazepam model. To facilitate interpretation of these findings, Figure 6 summarizes the proposed pharmacological mechanisms linking the three experimental models to α5-containing GABA_A_ receptor modulation. The scheme integrates the behavioral findings of the present study with previously published evidence and is intended as a conceptual framework rather than direct mechanistic proof.

Because the present study was intentionally designed as a comparative behavioral pharmacology study, the proposed mechanisms should be interpreted as biologically plausible hypotheses supported by previously published evidence rather than as experimentally demonstrated mechanisms. Consequently, the present findings should be interpreted within the limits of behavioral observations and considered as a foundation for future studies integrating molecular, electrophysiological, and histological approaches.

The most pronounced effects were observed in the diazepam-induced memory impairment model, providing further evidence that α5-containing GABA_A_ receptors contribute to the amnestic effects of benzodiazepines [36]. These observations support the rationale for selective α5 negative allosteric modulation as a strategy to reduce benzodiazepine-associated cognitive adverse effects while preserving the therapeutic actions mediated by other receptor subtypes [14].

The findings observed in the THP model were less pronounced. Although THP reduced spatial memory performance, basmisanil produced only limited recovery. These findings suggest that cholinergic dysfunction may involve additional neurobiological mechanisms beyond α5-containing GABA_A_ receptor modulation [3,37,38].

In the LPS model, basmisanil was associated with partial improvement in spatial memory performance, reflected by a significant interaction in the factorial analysis and an intermediate estimated recovery effect. However, the formal cross-model comparison did not demonstrate statistically significant differences in recovery effects, and these findings should therefore be interpreted with appropriate caution. These findings are consistent with the concept that neuroinflammation may alter inhibitory neurotransmission indirectly rather than through primary α5 receptor dysfunction [39,40,41,42].

Across the three experimental models, basmisanil administered alone produced no enhancement of baseline Y-maze performance. This finding suggests that its behavioral effects are more likely to reflect attenuation of specific pathological mechanisms underlying memory impairment than a generalized procognitive action in healthy animals.

Taken together, the present findings suggest that the contribution of α5-containing GABA_A_ receptors to cognitive dysfunction is mechanism-dependent rather than universal. Accordingly, the therapeutic potential of selective α5 negative allosteric modulation appears to depend on the neurobiological mechanism underlying memory impairment. Selective α5 negative allosteric modulators appear particularly promising for reducing benzodiazepine-induced amnesia, whereas their therapeutic potential in cholinergic dysfunction and neuroinflammation requires further investigation [7,43,44,45].

### Limitations

The present study has several methodological strengths but also limitations that should be considered when interpreting the findings. By applying the same behavioral tests, experimental design, and statistical framework across three mechanistically distinct models of memory impairment, it enabled a direct comparison of the effects of α5 GABA_A_ receptor modulation under different pathological conditions. The use of factorial analyses together with estimated marginal means further allowed assessment of both the independent and interactive effects of basmisanil in each experimental model, providing a more comprehensive evaluation than simple between-group comparisons. Nevertheless, several limitations should be acknowledged. First, the relatively small sample size (*n* = 7 per group) may have limited the statistical power to detect more subtle differences between experimental groups. Second, spatial memory performance was evaluated using a single behavioral test (Y-maze), which primarily assesses hippocampal-dependent spatial recognition memory. Additional behavioral tests targeting complementary domains of cognition, such as the Morris water maze or novel object recognition test, would provide a more comprehensive assessment of cognitive function. Third, only a single dose of basmisanil was evaluated, preventing assessment of potential dose-dependent effects.

Finally, the present study relied exclusively on behavioral outcomes and did not include complementary molecular, electrophysiological, or histological analyses to confirm receptor-specific mechanisms or quantify the degree of neuroinflammation in the LPS model. Furthermore, the study was intentionally designed as a comparative behavioral pharmacology study using an identical experimental protocol across all models. Although additional behavioral paradigms could provide complementary information on other cognitive domains, the standardized Y-maze protocol was considered essential for direct comparison of pharmacological effects across distinct mechanisms of memory impairment. Accordingly, the mechanistic interpretations proposed in the present study should be regarded as hypothesis-generating rather than definitive mechanistic evidence and require confirmation through future studies integrating behavioral, molecular, electrophysiological, and histological approaches.

## 5. Conclusions

The present findings suggest that basmisanil produced the clearest and most robust attenuation of spatial memory impairment in the diazepam model, providing further behavioral evidence consistent with the proposed contribution of α5-containing GABA_A_ receptors to the amnestic effects of benzodiazepines. An improvement in spatial memory performance was also observed in the LPS model, although it appeared less robust across complementary analyses, whereas no significant recovery was detected in the THP model. Formal comparison did not demonstrate statistically significant differences in the magnitude of the basmisanil effect across the three experimental models.

Overall, these findings suggest that the contribution of α5-containing GABA_A_ receptors to cognitive dysfunction is mechanism-dependent rather than universal. Basmisanil produced the most pronounced attenuation of spatial memory impairment in the diazepam model, showed partial improvement in the LPS model, and had no significant effect in the THP model. Although formal cross-model comparisons did not demonstrate statistically significant differences in recovery effects, the observed behavioral pattern is consistent with the hypothesis that selective α5 negative allosteric modulation may represent a promising strategy for mitigating benzodiazepine-associated cognitive impairment while indicating that its therapeutic potential in other forms of cognitive dysfunction requires further investigation. These findings further emphasize the importance of considering disease-specific neurobiological mechanisms when evaluating novel cognitive enhancers targeting GABA_A_ receptor signaling.

## Figures and Tables

**Table 1:** Effects of diazepam, basmisanil, and their combination on exploratory activity during the acquisition phase (T1).

Group	Time in O Arm (s)	Entries in O Arm
Control	119.0 ± 32.2	6.6 ± 1.0
Diazepam	117.1 ± 18.6	4.6 ± 0.5
Basmisanil	124.9 ± 27.2	7.3 ± 1.5
Diazepam + Basmisanil	105.1 ± 17.0	4.6 ± 0.8

Data are expressed as mean ± SD (*n* = 7 rats/group).

**Table 2:** Two-way factorial analysis of the primary outcome (Time Index) in the diazepam model.

Effect	df1	df2	F	*p*	Partial η^2^
Diazepam	1	24	1.71	0.203	0.067
Basmisanil	1	24	13.49	0.001	0.360
Diazepam × Basmisanil	1	24	19.79	<0.001	0.452

Note. Partial η^2^ = partial eta squared.

**Table 3:** Estimated marginal means for Time Index in the diazepam model.

Group	*n*	EMM	SE	95% CI
Control	7	50.87	1.97	46.80–54.95
Diazepam	7	39.51	1.97	35.43–43.58
Basmisanil	7	49.34	1.97	45.27–53.42
Diazepam + basmisanil	7	55.54	1.97	51.47–59.62

Note. EMM = estimated marginal mean; SE = standard error.

**Table 4:** Effects of THP, basmisanil, and their combination on exploratory activity during the acquisition phase (T1).

Group	Time in O Arm (s)	Entries in O Arm
Control	164.4 ± 73.0	4.6 ± 1.4
THP	126.7 ± 59.9	5.7 ± 2.1
Basmisanil	107.1 ± 48.5	5.9 ± 1.9
THP + Basmisanil	116.1 ± 61.5	5.0 ± 1.8

Data are expressed as mean ± SD (*n* = 7 rats/group).

**Table 5:** Two-way factorial analysis of the primary outcome (Time Index) in the THP model.

Effect	df1	df2	F	*p*	Partial η^2^
THP	1	24	8.02	0.009	0.251
Basmisanil	1	24	0.09	0.772	0.004
THP × basmisanil	1	24	0.68	0.419	0.027

**Table 6:** Estimated marginal means for Time Index in the THP model.

Group	*n*	EMM	SE	95% CI
Control	7	53.29	4.63	43.73–62.85
THP	7	36.35	4.63	26.79–45.92
Basmisanil	7	50.84	4.63	41.27–60.40
THP + basmisanil	7	41.52	4.63	31.96–51.08

Note. EMM = estimated marginal mean; SE = standard error.

**Table 7:** Effects of LPS, basmisanil, and their combination on exploratory activity during the acquisition phase (T1).

Group	Time in O Arm (s)	Entries in O Arm
Control	110.1 ± 15.6	5.3 ± 1.8
LPS	102.1 ± 25.0	5.3 ± 1.1
Basmisanil	98.6 ± 12.2	5.7 ± 1.5
LPS + Basmisanil	97.9 ± 21.7	5.9 ± 2.4

Data are expressed as mean ± SD (*n* = 7 rats/group).

**Table 8:** Two-way factorial analysis of the primary outcome (Time Index) in the LPS model.

Effect	df1	df2	F	*p*	Partial η^2^
LPS	1	24	2.30	0.142	0.088
Basmisanil	1	24	0.59	0.448	0.024
LPS × basmisanil	1	24	6.91	0.015	0.224

Note. Partial η^2^ = partial eta squared.

**Table 9:** Estimated marginal means for Time Index in the LPS model.

Group	*n*	EMM	SE	95% CI
Control	7	53.58	4.18	44.95–62.22
LPS	7	36.24	4.18	27.61–44.87
Basmisanil	7	45.81	4.18	37.18–54.44
LPS + basmisanil	7	50.46	4.18	41.83–59.09

Note. EMM = estimated marginal mean; SE = standard error.

**Table 10:** Recovery effects of basmisanil across the three experimental models.

Model	Δ	SE	95% CI	t	df	*p*	Holm-Adjusted *p*	Hedges’ g
Diazepam	16.04	6.51	[2.82, 29.25]	2.46	36	0.019	0.056	2.17
THP	5.17	6.51	[−8.05, 18.38]	0.79	36	0.433	0.433	0.32
LPS	14.22	6.51	[1.01, 27.43]	2.18	36	0.036	0.071	1.01

Note. Δ represents the estimated recovery effect of basmisanil, calculated as the mean difference between the *agent + basmisanil* group and the corresponding *agent alone* group. Holm-adjusted *p* values were corrected for the three simple-effect comparisons.

**Table 11:** Planned contrasts between control and inducing agent groups for Time Index U.

Model	Contrast	Δ	95% CI	Holm-Adjusted *p*	Hedges’ g
Diazepam	Control vs. Diazepam	−11.37	[−17.13, −5.60]	<0.001	−1.86
THP	Control vs. THP	−16.94	[−30.46, −3.41]	0.049	−1.39
LPS	Control vs. LPS	−17.34	[−29.55, −5.13]	0.022	−1.65

Note. Negative values indicate a reduction in Time Index U relative to the corresponding control group.

## Data Availability

Data are contained within the article.

## Abbreviations

The following abbreviations are used in this manuscript:

THP	Trihexyphenidyl
LPS	Lipopolysaccharide
